# Research on gearbox temperature field image fault diagnosis method based on transfer learning and deep belief network

**DOI:** 10.1038/s41598-023-33858-w

**Published:** 2023-04-24

**Authors:** Xi Lu, Pan Li

**Affiliations:** School of Mechanical Engineering, Southeast University, Nanjing, 211189 China

**Keywords:** Aerospace engineering, Mechanical engineering

## Abstract

This paper applies thermal imaging technology to gearbox fault diagnosis. The temperature field calculation model is established to obtain the temperature field images of various faults. A deep learning network model combining transfer learning of convolutional neural network with supervised training and unsupervised training of deep belief network is proposed. The model requires one-fifth of the training time of the convolutional neural network model. The data set used for training the deep learning network model is expanded by using the temperature field simulation image of the gearbox. The results show that the network model has over 97% accuracy for the diagnosis of simulation faults. The finite element model of gearbox can be modified with experimental data to obtain more accurate thermal images, and this method can be better used in practice.

## Introduction

Vibration signal monitoring is the most commonly used method for gearbox fault diagnosis. Vibration signal monitoring has the advantages of moderate cost, strong reliability and mature technology^1^. The defects of this method include contact measurement, local information, serious influence of environmental conditions, serious loss of long-distance transmission signal caused by noise pollution^2,3^. It is an important supplement to the basis of fault determination that the introduction of temperature signal into the field of gearbox fault diagnosis.

Recent studies have shown that the temperature signal contains a large amount of information that can be used for gearbox health status detection and fault diagnosis^4^. Infrared thermal imaging technology which has the advantages of more comprehensive information and no contact in temperature measurement is more concerned by researchers^5^. Kwan et al. developed a neural network-based image processing tool that can detect abnormal temperature increases five hours before tooth fracture^6^. Younus et al. proposed a new method for fault diagnosis of rotating motors based on thermal image research using image histogram features. It is proved that the classification process of thermal image features by classifiers such as support vector machine can serve machine fault diagnosis^7^. Subsequently, they proposed an intelligent diagnostic system to classify different machine states using infrared thermal imaging^8^. Lim et al. compared thermal images with vibration signals and proposed a fault diagnosis method using support vector machine algorithm through infrared thermal imaging^5^. Emmanuel Resendiz-Ochoa et al. proposed a method for diagnosing gear wear by analyzing infrared imaging images. The method firstly calculates the statistical time domain characteristics of infrared imaging, then reduces the dimension of data, and finally performs fault diagnosis by neural network^9^. Most of these studies are fault diagnosis under a specific condition. On this basis, Shao et al. proposed a transfer learning method using convolutional neural networks for bearing fault diagnosis under different operating conditions^10^. Yongbo Li et al. extracted fault features from thermal imaging images by using the bag-of-visual-words method, and then classified rotating machinery faults by using support vector machine, realizing the fault diagnosis of rotating machinery under non-stationary operating conditions^11^. Bai Tangbo proposed a fault diagnosis method for rotating machinery that can solve the shortcomings of low contrast, blurred edges and high noise of infrared thermal images^12^.

The above research has a common difficulty in improving accuracy, that is, the data set used for training and verification is small, and it is difficult to obtain all data under various fault conditions^3^.

In this paper, the finite element model of gearbox is established, the temperature field distribution on the surface of steady-state gearbox is calculated, and the corresponding temperature field image is obtained. A deep learning network model for gearbox fault diagnosis is proposed, which combines the transfer learning of convolutional neural network with deep belief network. It is verified that the gear box temperature field image has a high accuracy rate for gearbox fault diagnosis.

## Create CAE model

**Figure 1:**
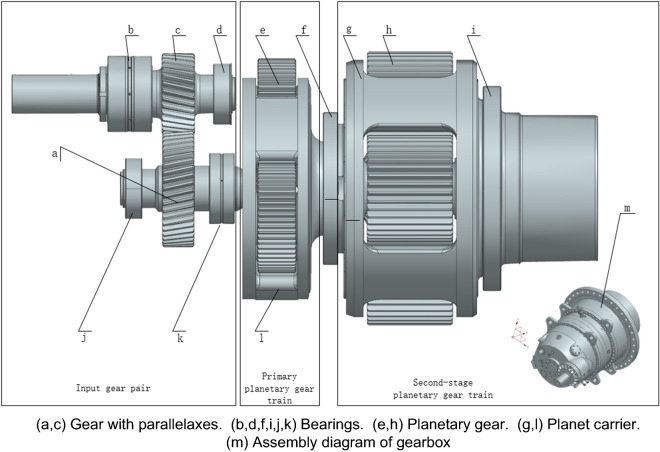
Gearbox three-dimensional model.

The gearbox used in this paper is shown in Fig. 1, which is composed of an input gear pair with parallelaxes and two sets of planetary gear trains. The gear parameters are shown in Table 1.

**Table 1:** Gear parameters.

	Input gear pair		Primary planetary gear train			Second-stage planetary gear train		
	Driving gear	Driven gear	Sun gear	Planetary gear	Ring gear	Sun gear	Planetary gear	Ring gear
Module	9	9	7	7	7	10	10	10
Number of teeth	32	46	24	57	138	24	38	100

### Gearbox heating power calculation

The heat transfer modes include heat conduction, heat convection and heat radiation. The relative rolling and sliding of the gear, the rolling of the bearing, the power loss caused by the gear and bearing mixing oil and mixing gas are the main sources of heat generated by the gearbox in the working state.

There are many formulas for calculating the power loss of gears. In this paper, Anderson-Loewenthal calculation method is used^13^. The Friction torque calculation formula of low speed bearing of Palmgren is selected to calculate the heat production power of the bearing^14^. The mechanical design manual is used to calculate the total efficiency of the planetary gear train to calculate the total power loss^15^. The churning loss of shaft and the churning loss of gear and bearing on both sides are calculated by the British standard BS ISO/TR14179-1-2001^16^. The formulas selected above are given in Table 2.

**Table 2:** Calculation formula of heat production of gearbox.

Gear pair with parallelaxes	Heat-generating power of gear tooth surface	Rolling loss	$P_{r} = C_{1} V_{r} F_{R}$
		Sliding loss	$$P_{s} = C_{1} F_{S} V_{s} $$
		Windage loss	$P_{w,g} = C_{2} \left( {1 + 2. 3\frac{b}{R}} \right)\left( {\frac{n}{{m_{g} }}} \right)^{2. 8} R^{4. 6} \left( {0. 28\mu + C_{3} } \right)^{0. 2}$ $P_{w,p} = C_{2} \left( {1 + 2. 3\frac{b}{R}} \right)n^{2. 8} R^{4. 6} \left( {0. 28\mu + C_{3} } \right)^{0. 2}$
		Oil stirring power loss	$C_{h} = \frac{1}{2}\rho \omega^{2} S_{m} \left( {D_{p} /2} \right)^{3} C_{m}$ $C_{m} = \varphi_{1} \left( {\frac{m}{{D_{p} }}} \right)^{{\varphi_{2} }} \left( {\frac{b}{{D_{p} }}} \right)^{{\varphi_{3} }} \left( {\frac{{h_{m} }}{{D_{p} }}} \right)^{{\varphi_{4} }} \left( {\frac{{V_{0} }}{{D_{p}^{3} }}} \right)^{{\varphi_{5} }} Re^{{\varphi_{6} }} Fr^{{\varphi_{7} }}$ $P_{G} = nC_{h} /9549$
	Heat-generating power of gear(bearing) both side stirring oil		$P = \frac{{1.474f_{g} vn^{3} D^{5.7} }}{{A_{g} \times 10^{26} }}$
Bearing friction heat generating power			$M_{l} = f_{l} P_{l} d_{m}$ $$M_{v} = \left\{ {\begin{array}{*{20}c} {10^{ - 7} f_{0} \left( {vn} \right)^{2/3} d_{m}^{3} vn \ge 2000 } \\ {160 \times 10^{ - 7} f_{0} d_{m}^{3} vn < 2000} \\ \end{array} } \right.$$ $M = M_{l} + M_{v}$ $P =\uppi {\text{nM}}/60$
Calculation of planetary gear train efficiency			$\eta_{AX}^{B} = \eta_{XA}^{B} = 1 - \frac{{\psi^{X} }}{{1 + \left| {i_{BA}^{X} } \right|}}$ $\eta_{BX}^{A} = \eta_{XB}^{A} = 1 - \frac{{\psi^{X} }}{{1 + \left| {i_{AB}^{X} } \right|}}$ $\eta_{AB}^{X} = \eta_{BA}^{X} = 1 - \psi^{X}$
Heat-producing power of shaft			$P = \frac{{7. 37f_{g} vn^{3} D^{4. 7} L}}{{A_{g} \times 10^{26} }}$

The heat transfer coefficient of different surfaces of the gearbox varies with the contact medium. The convective heat transfer coefficient (CHTC) of the gear tooth surface adopts the mathematical model proposed by Handschuh to calculate CHTC of the gear meshing surface. CHTC of the two sides of the gear and the planet carrier adopts the calculation formula of CHTC of the rotating disk and the medium^17^. CHTC of bearing and shaft is calculated by the formula in Zhao's paper^18^. CHTC between the inner surface of the box and the medium is based on the Gnielinski formula, and CHTC between the outer surface and the medium is based on the single tube criterion proposed by Churchill and Bernstein^19^. The formulas selected above are given in Table 3.

**Table 3:** Heat transfer coefficient calculation formula of gearbox.

Gear	Gear tooth surface heat transfer coefficient	$h = 0. 228Re^{0. 731} Pr^{0. 333} k/L$
	Gear side (planet carrier)heat transfer coefficient	$h = 0. 0197km_{e} + 2. 6^{0. 2} Pr^{0. 6} \left( {\frac{\omega }{v}} \right)^{0. 2} r^{0. 6}$
Gearbox	Heat transfer coefficient of gearbox inner surface	$Nu_{f} = \frac{{\left( {{\text{f}}_{{\text{c}}} /8} \right)\left( {Re - 1000} \right)Pr_{f} }}{{1 + 12. 7\sqrt {\left( {\frac{{{\text{f}}_{{\text{c}}} }}{8}} \right)} \left( {Pr_{f}^{2/3} - 1} \right)}}\left[ {1 + \left( \frac{d}{l} \right)^{2/3} } \right]c_{t}$
	Heat transfer coefficient of external surface of gearbox	$h = 0. 3 + \left\{ {\left( {0. 62Re^{1/2} Pr^{1/3} } \right)/\left[ {1 + \left( {0. 41/Pr} \right)^{2/3} } \right]^{1/4} } \right\}$ $\left[ {1 + \left( {\frac{Re}{{282000}}} \right)^{5/8} } \right]^{4/5}$
Bearing		$h = 0. 332kPr^{1/3} \left( {\frac{{\frac{1}{3}v_{s} }}{{vd_{m} }}} \right)^{1/2}$
Axis		$h = 0. 11\left( {0.{ }5Re^{2} Pr} \right)^{0. 35} \lambda /d_{a}$

The parameters used to establish the steady-state temperature field model of the gearbox can be obtained from the above formula.

### Establish steady-state thermal calculation model of gearbox

During normal operation, the temperature field of gearbox is gradually stable. The stable temperature field changes when the gear fails. The running state of the gearbox can be reflected by the temperature field distribution of the gearbox.

The calculation formula of steady-state temperature field model parameters is introduced above. The temperature field distribution on the surface of the gearbox can be obtained by establishing the calculation model of the gearbox from these parameters. The model is simplified as follows:

1. In the working state, the contact parts of the input gear pair are alternately contacted, and the heat generation is uniform. Therefore, the gear meshing part can be simplified. The calculated heat generation can be uniformly applied to the contact surface corresponding to the simplified gear model.
2. The meshing parts corresponding to each gear of the planetary gear train contact alternately, so the distribution of heat on the contact surface is uniform. Therefore, the simplification of the planetary gear train can be divided into the following three steps : First, calculate the overall heat flux of the gearbox. Then the planetary gear, sun gear and planet carrier of the model are removed to obtain the equivalent model. Finally, the heat flux density which transfer to shaft and the gear ring is uniformly applied to the corresponding position of the equivalent model.
3. Due to the high temperature of the gearbox in the input part and the first-stage planetary gear train, the characteristics of the temperature field distribution are obvious. The temperature of the output part (the second-stage planetary gear train part) is low, so the temperature field distribution characteristics are not obvious. Therefore, this paper only studies the gear fault diagnosis of the input part of the gearbox and the first-stage planetary gear train.

## Establish a deep learning network model

In this paper, the convolutional neural network model is first established. Then, a deep learning network model for gearbox fault diagnosis combining transfer learning and deep belief network is proposed, and the two models are compared.

### Introduction of fault diagnosis model

**Figure 2:**
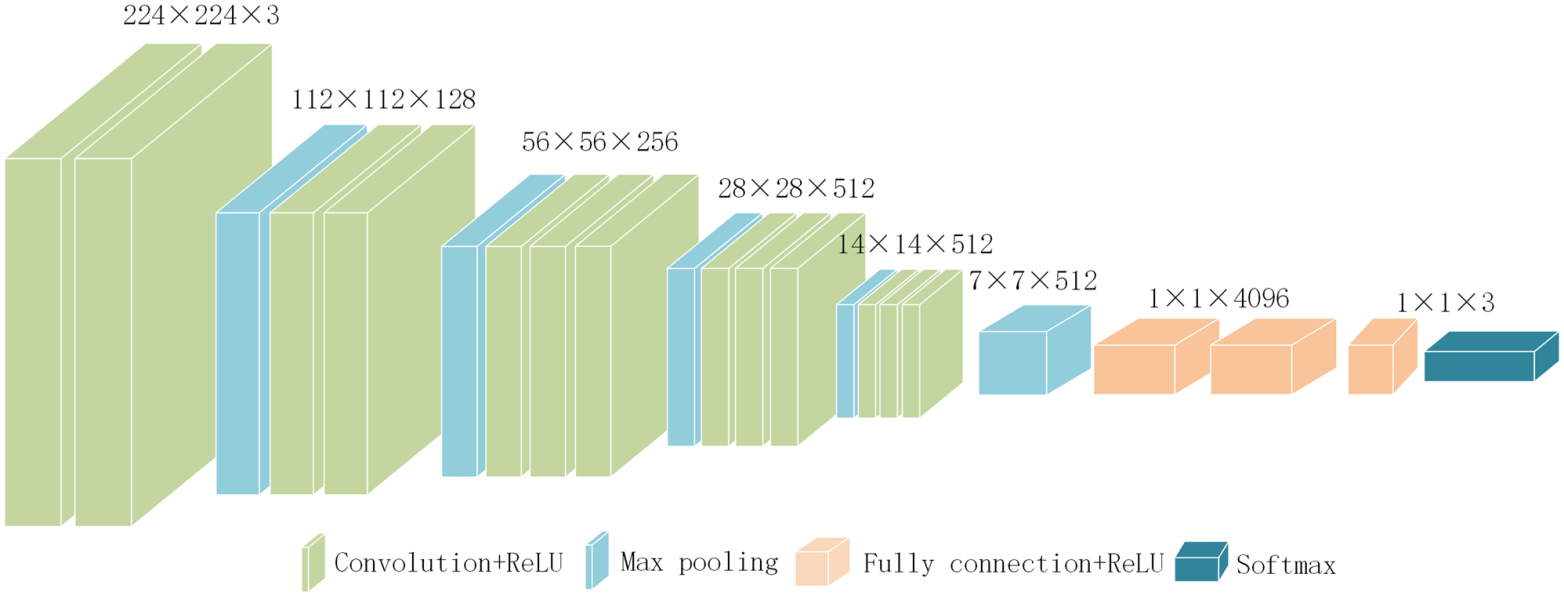
The structure of convolution network model.

Convolutional neural networks are widely used in image classification. Firstly, the convolutional network model is introduced. The structure of the model is shown in Fig. 2. In this paper, the convolutional neural network is used to train the data set to obtain the classification results.

The deep learning network model of gearbox fault diagnosis combining transfer learning of convolutional neural networks and deep belief network (TrCNN-DBN) consists of three parts, temperature field image expansion(TFIE), convolutional neural network transfer learning (TrCNN) and deep belief network (DBN) fault classification.

TFIE rotates the input image, adjusts the brightness of the image, adds noise and blur to the image. Rotation simulates the thermal imager jitter. Adjusting the brightness of the picture, adding noise and blurring the picture are used to simulate the changes in the light of the shooting environment. Image expansion can greatly increase the data set used in training, thereby improving accuracy.

**Figure 3:**
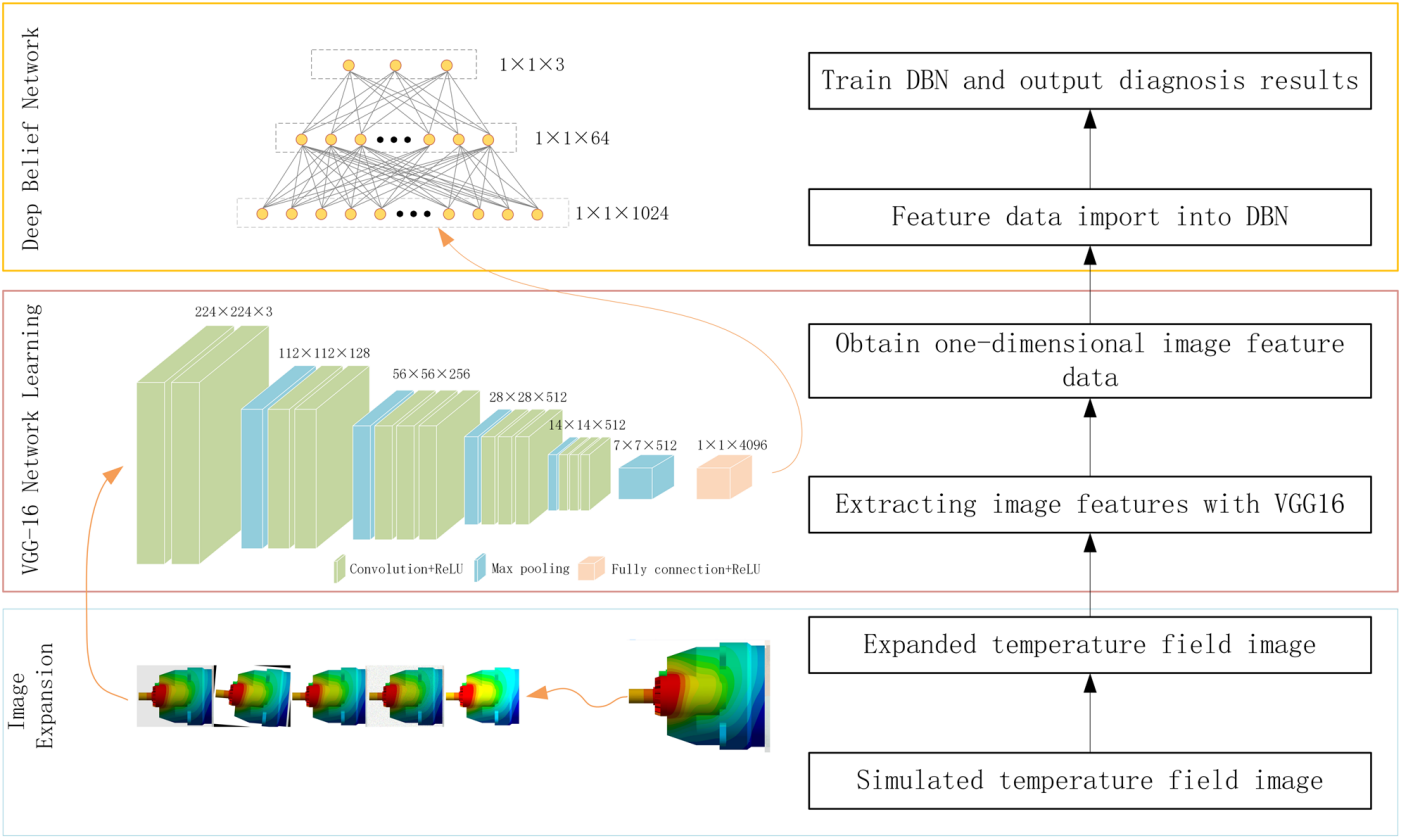
The structure of TrCNN-DBN.

TrCNN uses the VGG16 model proposed by the Visual Geometry Group at Oxford University. In this paper, the first 19-layer convolutional neural network of the VGG16 model is used to extract the features of the image. Then the obtained data is used for the training and classification of the DBN network. The model structure is shown in Fig. 3.

DBN is a probability generation model composed of Restricted Boltzmann Machine and Sigmoid Belief Network. The combination of supervised learning and unsupervised learning can better inherit the information of the upper layer of the network to the next layer compared with the fully connected layer of the convolutional neural network.

The diagnosis process is as follows:

1. The temperature field distribution images under specific working conditions simulated by the steady-state thermal calculation model of the gearbox are divided into two categories, namely the training set and the verification set.
2. The image of the training set is expanded to increase the number of samples in the training set and simulate images in different shooting environments.
3. The training set image with image enhancement and the verification set image are input into TrCNN for feature extraction.
4. The extracted feature data is input into DBN to further extract features for fault prediction.

**Figure 4:**
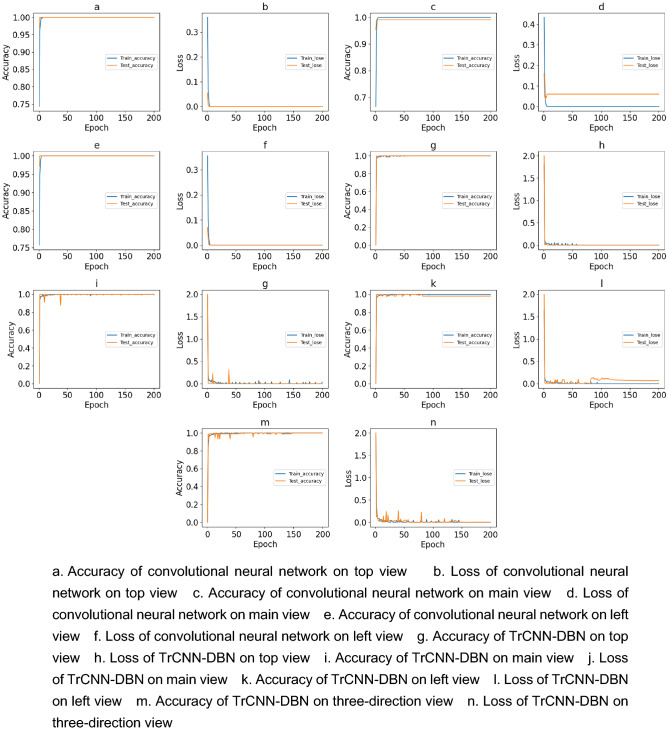
Training and testing performance curves.

As shown in Fig. 4, using convolutional neural network model to train the top view picture and the left view picture, the classification accuracy of the model reaches 100% after 3 epochs, and the loss is almost 0. The model classification accuracy of the main view picture trained with convolutional neural network model is 99%, and the loss is 0. 06. The classification accuracy of TrCNN-DBN on the top view picture and the main view picture is 100%, and the classification accuracy on the left view picture is 97%. The classification accuracy of the two models is similar, but the training time of convolutional neural network model is 5 times that of TrCNN-DBN. Therefore, the TrCNN-DBN model is selected for gearbox fault diagnosis, and the training results of the model TrCNN-DBN are introduced in detail.

### Three directions temperature field diagnosis

The steady-state temperature field distribution of the gearbox housing can be completely represented by the thermal images in three directions. Therefore, the thermal images in three directions are respectively enhanced and extracted, and then the obtained feature data is combined into a vector for training and fault prediction of DBN. The specific process is as follows :

It is assumed that the rotational speed, load, ambient temperature and the kinematic viscosity of the lubricating oil will not change when the gearbox works under the specificworking condition of the input shaft speed 1450 $\mathrm{r}/\mathrm{min}$, with the ambient temperature 20 $\mathrm{^\circ{\rm C} }$. Assuming that the heat production of a single heat generating component is higher than 20% of the normal situation, the fault diagnosis model gives an early warning of possible faults of the gearbox. The working conditions of the gearbox are shown in Table 4.

**Table 4:** Gearbox working environment.

Ambient temperature	Input speed	Output speed	Input power
20 °C	1450 ${\text{r}}/{\text{min}}$	20 ${\text{r}}/{\text{min}}$	900 ${\text{kW}}$
Rated output torque	Lubricating oil volume	Lubricating oil kinematic viscosity	lubricating system
1100 ${\text{kN}} \cdot {\text{m}}$	150 ${\text{L}}$	240 ${\text{mm}}^{2} /s$	Oil injection

Based on these two assumptions, the surface temperature field distribution of the gearbox housing is calculated when the heat production of each heat producing part is between 100 and 120% of the normal heat production. It is considered that the gearbox is in normal working condition at this time, and the surface temperature field image of the gearbox housing at this time is obtained. Then calculate the surface temperature field distribution of the gearbox when the heat production of a heat production part is between 120 and 140% of the heat production under normal conditions and the heat production of other heat production parts is under normal conditions. It is considered that the gearbox is in the fault state of the heat production part at this time, and the surface temperature field image of the gearbox at this time is obtained. The specific gradient can be selected according to the actual situation, and each calculation result is considered as a case. The obtained images are classified according to the training set and the verification set and operated according to the specific diagnosis process of the fault diagnosis model proposed above.

This paper simulates 150 cases of normal, 150 cases of fault 1(Part c in Fig. 1 fails) and 150 cases of fault 2(Part j in Fig. 1 fails). A total of 450 images of gearbox temperature field distribution were obtained, including 150 images under normal conditions, 150 images of fault 1 and 150 images of fault 2. Each one takes 125 pictures as the training set and 25 pictures as the verification set.

**Figure 5:**
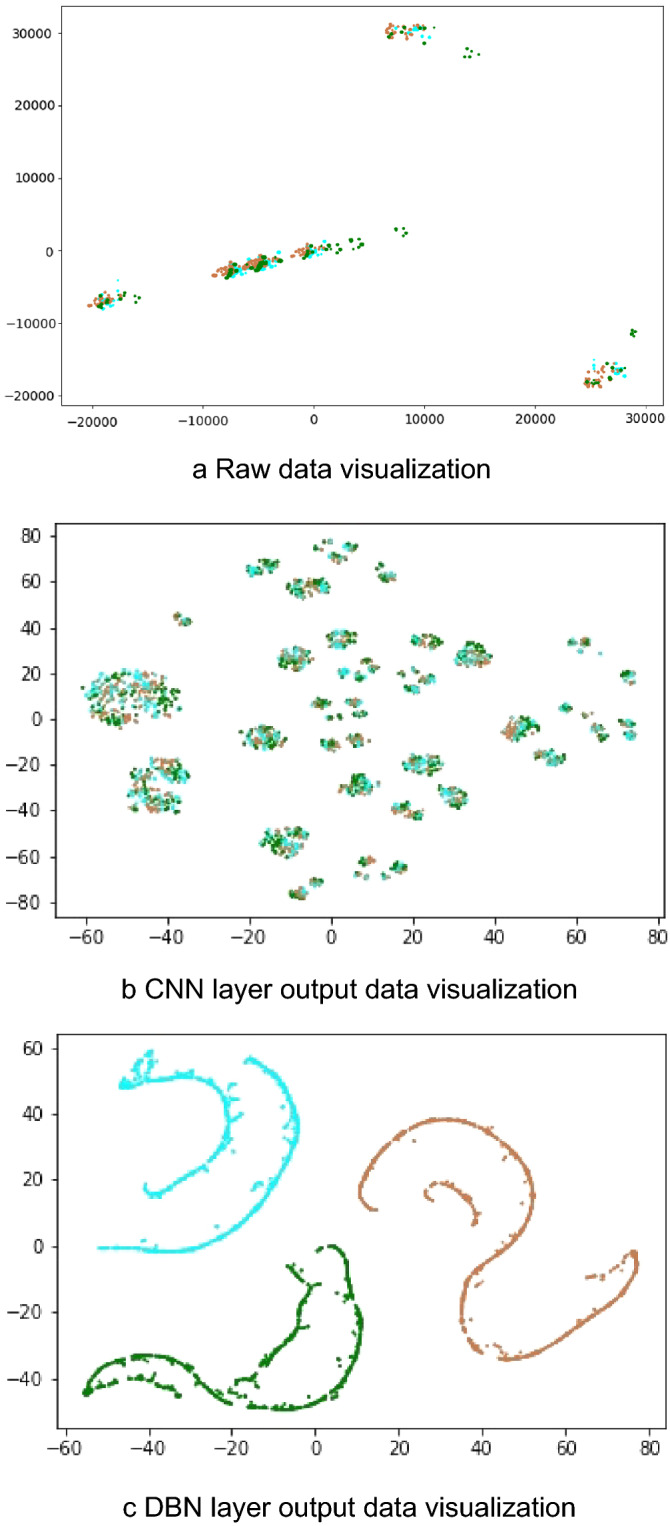
T-SNE dimensionality reduction visualization.

T-Distributed Stochastic Neighbor Embedding (t-SNE) is an unsupervised nonlinear technology, which is mainly used for data exploration and visualization of high-dimensional data. The image input to the model, the output of the CNN layer, and the output of the DBN layer are visualized using the t-SNE method. As shown in Fig. 5, the various types of data in the original image overlap together, and different fault types have shown some separability after passing through the CNN layer. After passing through the DBN layer, each type of data is completely separated, indicating that the model has better fault characteristics.

**Figure 6:**
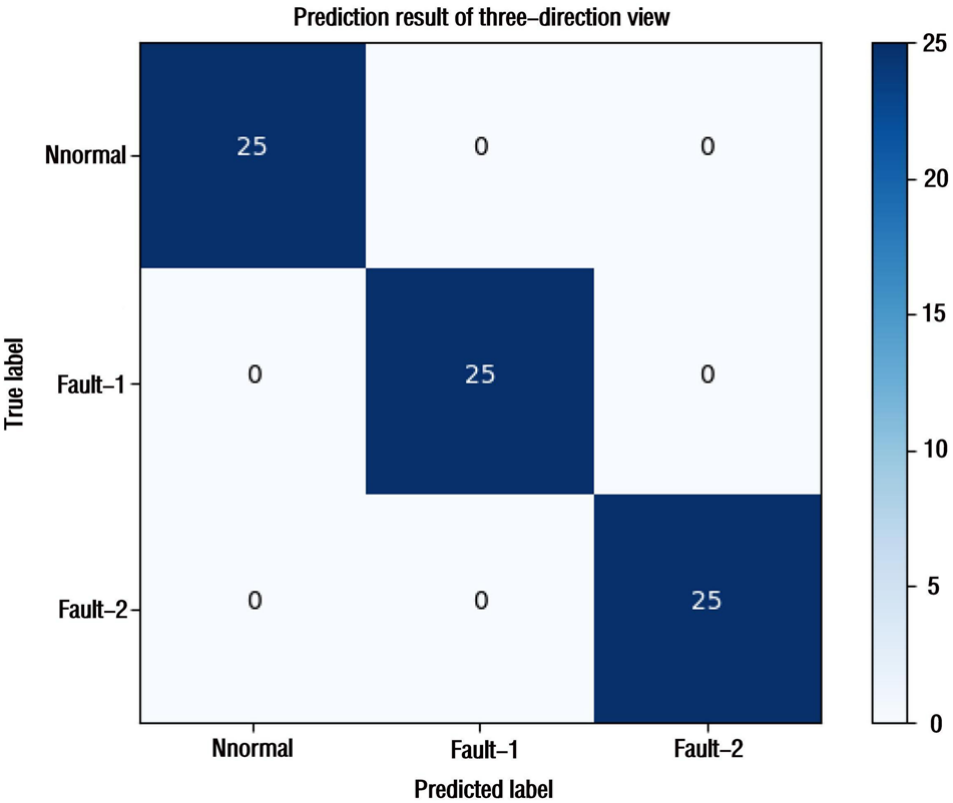
Confusion matrix of three-direction view.

**Figure 7:**
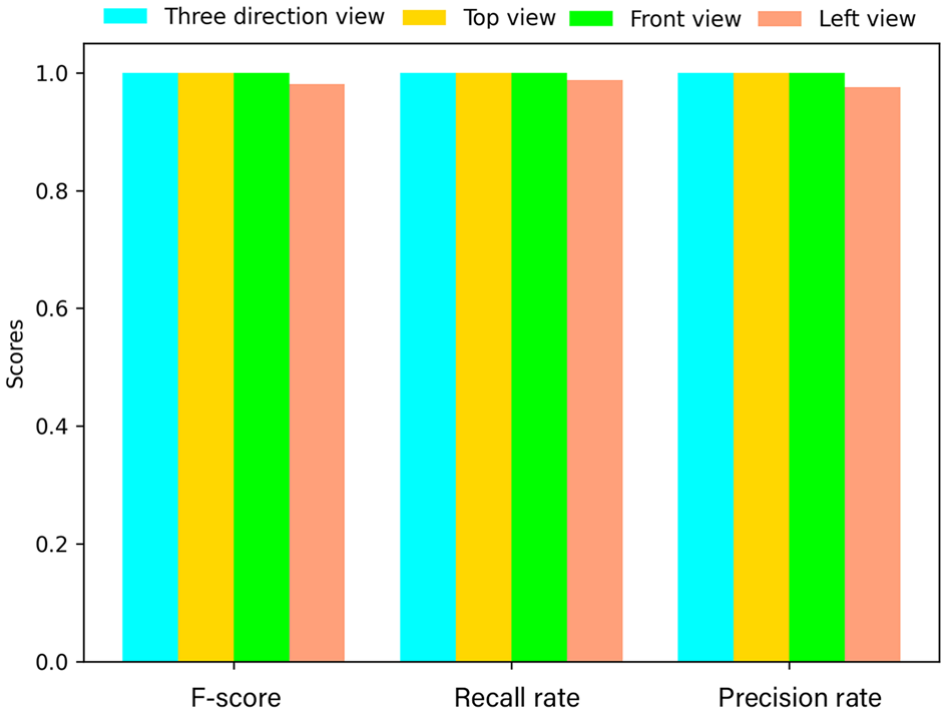
Precision rate,recall rate and F-score of the proposed method.

The final classification results of the model are shown in Fig. 6. From the confusion matrix, it can be seen that the TrCNN-DBN model proposed in this paper has 100% classification accuracy for each simulation condition. And f-score value, the recall rate and the precision rate of each working condition are 1, as shown in Fig. 7. The relationship between the accuracy of the model, the loss and the number of iterations is shown in Fig. 4m, n. It can be seen that after 150 epochs, the loss of the model on the training set and the verification set is reduced to close to 0. The accuracy is also 100% and almost unchanged. TrCNN-DBN proposed in this paper has high fault diagnosis accuracy for the steady-state temperature field distribution image of gearbox simulated by finite element.

### Single direction temperature field diagnosis

In practical work, on the one hand, some parts of the gearbox are more prone to failure than other parts. When a fault occurs, the whole gearbox will be detected,so those parts that are not prone to failure can be maintained during regular testing. The thermal imaging equipment is more expensive. In order to reduce the cost, it is necessary to reduce the number of thermal imaging equipment. On the other hand, due to the installation position of the gearbox, it may not be able to get three-direction thermal images. Therefore, this paper proposes the use of single-direction thermal image for fault diagnosis.

**Figure 8:**
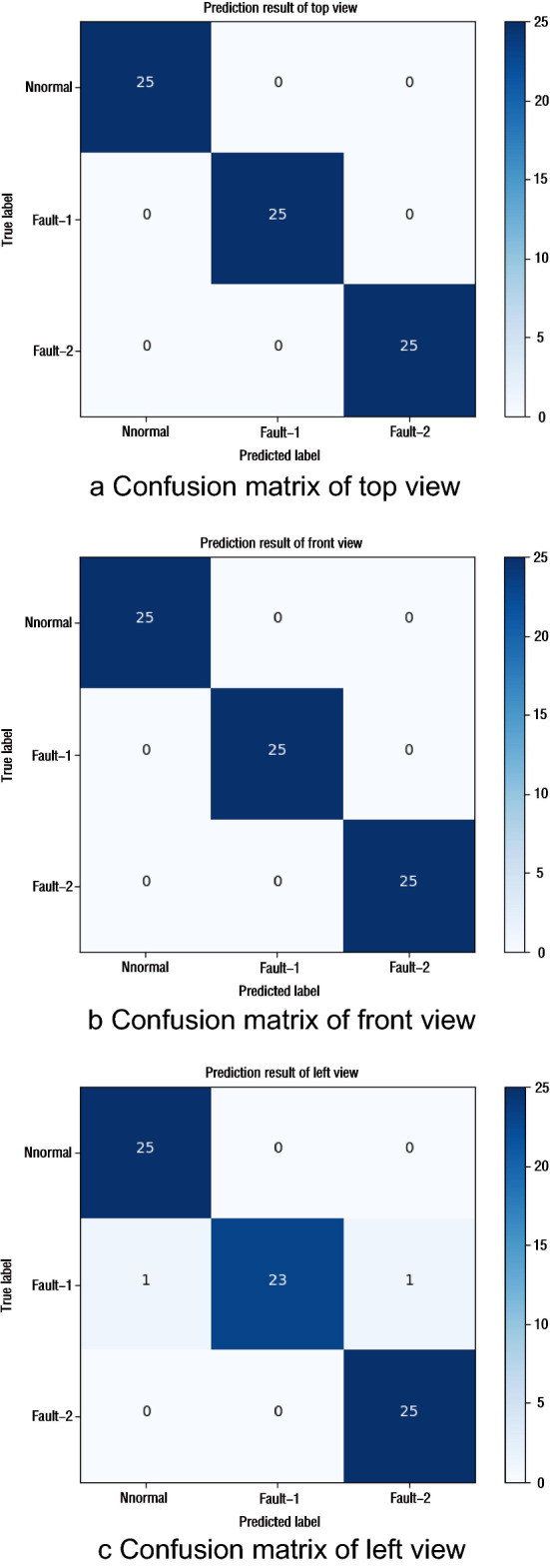
Confusion matrix of single-direction view.

The fault diagnosis is carried out by using the temperature field picture in one of the three directions intercepted above and the fault diagnosis model proposed above. The calculation results are shown in Fig. 8. As can be seen from the confusion matrix, the classification accuracy of the model on the top view and the main view is 100%, but the accuracy on the left view is 97%.

It can be found from Fig. 4 that the accuracy of the model on the top view has been 100% and stabilized after 60 epochs. Accuracy on the main view is close to 100% after 100 epochs, but still fluctuates. The accuracy on the left view stabilized at 97% after 100 epochs. And Fig. 7 also shows that the accuracy of the model for left view fault identification is lower than that of the other two directions. The above results show that the top view direction is the temperature sensitive direction of the two simulated faults, and fault diagnosis in this direction will have higher accuracy and stability. Comparing the results with the calculation results of the three directions images, it is found that the data of the fault insensitive direction will affect the classification results of the sensitive direction.

## Conclusion

In this paper, the finite element temperature field simulation image of the gearbox is combined with deep learning to apply to the gearbox condition monitoring (FETFS). The accuracy of TrCNN-DBN and convolutional neural network models is compared, and the classification results of TrCNN-DBN are described in detail. It is verified that the training time required by TrCNN-DBN is one fifth of that of convolutional neural network model. Moreover, TrCNN-DBN has high fault judgment accuracy for both three-way thermal images and one-way thermal images. The FETFS method can greatly expand the temperature field distribution database when the gearbox fails. TrCNN-DBN applies the transfer learning of the convolutional neural network to the fault diagnosis of the gearbox, reducing the number of parameters that need to be trained and saving time and equipment costs. The results show that TrCNN-DBN is sensitive and reliable for the diagnosis of the simulated temperature field images in three directions of single fault of the gearbox, and the accuracy of fault identification under the set working conditions and fault conditions is above 97%. In addition, it is proved that the simulation images in a single direction can be used to diagnose faults for specific faults, and the accuracy can also reach reach more than 97%.

This paper verifies that the fault identification of gearbox can be well carried out by calculating the temperature field image of the model. Finite element model updating based on experimental results is a mature technology that has been widely used^20–23^. Therefore, the method proposed in this paper can be tested according to the application object, and more accurate pictures can be obtained by modifying the model. Therefore, this paper is of great significance for establishing the digital twin heat transfer model of gearbox.

### Consent to participate

The authors agree to the authorship order.

## Data Availability

The data that support the findings of this study are available from Nanjing High Speed Gear Manufacturing Co., Ltd. but restrictions apply to the availability of these data, which were used under license for the current study, and so are not publicly available. If you need to get data, you can contact the corresponding author of this article.

## List of symbols

*A*_*g*_: Configuration constant
b: Tooth face width, m
*c*_*t*_: Correction coefficient of temperature difference
*C*_(1,2,3)_: Constants of proportionality
*C*_*h*_: Drag moment coefficient of liquid agitation
*C*_*m*_: Drag torque coefficient
*d*: Diameter of gearbox, m
*d*_*a*_: Shaft diameter, m
*d*_*m*_: Bearing pitch diameter, m
*D*: Gear pitch diameter, m
*D*_*p*_: Pitch diameter, m
f_c_: Darcy resistance coefficient
*f*_0_: Coefficient related to
*f*_*g*_: Gear wetting factor
*f*_*l*_: Coefficient of load
Fr: Froude number
*F*_*R*_: Rolling traction force, N
*F*_*S*_: Slidling force, N
*h*: Convection heat transfer
*h*_*m*_: Immersion depth of gear in oil, m
i: Transmission ratio
*k*: Oil heat conductivity, W/(m K)
*l*: Gearbox length, m
*L*: Characteristic size, m
*m*: Module
*m*_*e*_: Exponential constant
*m*_*g*_: Gear ratio, N_g_/N_p_
*M*: Total friction torque, N/m
*M*_*l*_: Friction moment caused by load, N/m
*M*_*v*_: Bearing friction torque related to lubricating oil viscosity, N/m
*n*: Rotational speed, rpm
*Nu*_*f*_: Average Nusselt number
*P*: Power, KW
*P*_*l*_: Equivalent dynamic load, N
*P*_*r*_: Rolling lose, KW
*Pr*_*f*_: Average prandtl number
*P*_*s*_: Sliding loss, KW
*P*_*w*_: Windage loss, KW
*P*_*G*_: Oil stirring power loss, KW
*Pr*: Prandtl number
r: Radius of gear base circle, m
R: Gear pitch radius, m
*Re*: Reynolds number
*S*_*m*_: Submerged surface area, m^2^
*v*: Fluid kinematic viscosity, m^2^/s
*v*_*s*_: Bearing cage speed, m/s
*V*_*0*_: Lubricating oil volume, V
*V*_*r*_: Rolling speed, m/s
*V*_*s*_: Sliding velocity, m/s
*g*: Gear
*p*: Pinion
A: Solar gear
B: Gear ring
C: Planetary gear
X: Planet carrier
η: Efficiency
*λ*: Air heat conductivity, W/(m K)
*ρ*: Fluid density, kg/m^3^
*φ*_1__,2,3,4,5,6,7_: Constant coefficient obtained by experiment
*ω*: Rotational angular velocity, rad/min
*μ*: Lubricant absolute viscosity, P_a_ s
Ψ: Transmission loss coefficien
